# Activation of the heat shock response as a therapeutic strategy for tau toxicity

**DOI:** 10.1242/dmm.050635

**Published:** 2024-10-01

**Authors:** Taylor R. Stanley, Elizabeth M. Otero, Amy L. Knight, Aleen D. Saxton, Xinxing Ding, Melissa Borgen, Brian C. Kraemer, Karen S. Kim Guisbert, Eric Guisbert

**Affiliations:** ^1^ Biomedical Sciences Program, Florida Institute of Technology, Melbourne, Florida; ^2^ Geriatrics Research Education and Clinical Center (GRECC), Veterans Affairs Puget Sound Healthcare System, 1660 South Columbian Way Seattle, WA 98108-1532, USA; ^3^ Division of Gerontology and Geriatric Medicine, Department of Medicine, University of Washington Harborview Medical Center, 325 9th Ave, Box 359755, Seattle, WA 98104-2499, USA; ^4^ Department of Psychiatry and Behavioral Sciences, University of Washington, 1959 NE Pacific Street, Box 356560, Seattle, WA 98195-6560, USA; ^5^ Department of Laboratory Medicine and Pathology, University of Washington, 1959 NE Pacific Street, Box 357470, Seattle, WA 98195, USA; ^6^ Department of Biology, College of Arts and Sciences, University of Nebraska Omaha, Omaha, Nebraska

**Keywords:** Alzheimer’s disease, Tau (MAPT), HSF1, Heat shock response, *Caenorhabditis elegans*

## Abstract

Alzheimer's disease is associated with the misfolding and aggregation of two distinct proteins, beta-amyloid and tau. Previously, it has been shown that activation of the cytoprotective heat shock response (HSR) pathway reduces beta-amyloid toxicity. Here, we show that activation of the HSR is also protective against tau toxicity in a cell-autonomous manner. Overexpression of HSF-1, the master regulator of the HSR, ameliorates the motility defect and increases the lifespan of transgenic *C. elegans* expressing human tau. By contrast, RNA interference of HSF-1 exacerbates the motility defect and shortens lifespan. Targeting regulators of the HSR also affects tau toxicity. Additionally, two small-molecule activators of the HSR, Geranylgeranylacetone (GGA) and Arimoclomol (AC), have substantial beneficial effects. Taken together, this research expands the therapeutic potential of HSR manipulation to tauopathies and reveals that the HSR can impact both beta-amyloid and tau proteotoxicity in Alzheimer's disease.

## Teaser

**Summary:** Activation of the heat shock response both genetically and pharmacologically was found to ameliorate tau toxicity in *C. elegans*.

## INTRODUCTION

Alzheimer's disease (AD) is a debilitating neurodegenerative disease that causes a dramatic loss of neuronal cells (Long and Holtzman, 2019). AD patients exhibit extreme memory defects and substantial cognitive impairment. Each year, there are ∼10 million new cases of AD worldwide, accounting for 60−70% of all dementia cases. There is currently no cure for AD (Boxer and Sperling, 2023).

Two key pathological features in the brain of AD patients are amyloid plaques and neurofibrillary tangles (Selkoe and Hardy, 2016). Amyloid plaques form outside neurons and contain the beta-amyloid peptide. In contrast, neurofibrillary tangles form inside neuronal cells and contain the misfolded and hyperphosphorylated microtubule-binding protein (MAPT; also known as and, hereafter, referred to as tau). Tau protein aggregates are also associated with other neurodegenerative diseases, such as frontotemporal dementia with parkinsonism (FTDP), which are collectively known as tauopathies.

Protein misfolding and aggregation are common features of neurodegenerative diseases (Soto, 2003). For example, Lewy bodies found in Parkinson's disease patients consist of aggregated alpha-synuclein (SNCA), and inclusions associated with Huntington's disease contain aggregated huntingtin protein (HTT) containing expanded polyglutamine repeats (Ross and Poirier, 2004). Pathological protein aggregation is one of the eight major hallmarks of neurodegenerative disease (Wilson et al., 2023). Other hallmarks include aberrant proteostasis, cytoskeletal abnormalities, altered energy homeostasis, DNA and RNA defects, inflammation, synaptic and neuronal network dysfunction, and neuronal cell death. Strategies that target these hallmarks represent potential new therapeutic interventions.

Protein misfolding and aggregation can be prevented by activation of a cellular pathway known as the heat shock response (HSR) (Guisbert et al., 2008). The HSR is mediated by heat shock factor 1 (HSF1) transcription factor (Gomez-Pastor et al., 2018). The HSF1 regulon contains a set of molecular chaperones that bind directly to misfolded proteins, enhancing their refolding or promoting their degradation (Brunquell et al., 2016). The HSR also regulates other parts of the proteostasis network, a large collection of genes that influence protein synthesis, folding, trafficking and degradation, and collectively maintain protein-folding homeostasis (Balchin et al., 2016; Brehme and Voisine, 2016).

HSR activation has been validated as a therapeutic strategy for several neurodegenerative diseases (Klaips et al., 2018). The small molecule Arimoclomol (AC), a co-activator of the HSR, provides some of the most compelling data for this approach, as it has demonstrated efficacy in mouse models and in phase II clinical trials in patients with amyotrophic lateral sclerosis (ALS) (Benatar et al., 2018; Kalmar et al., 2008). With respect to AD, HSR activation has been shown to ameliorate beta-amyloid toxicity in disease models, but its relevance remains uncertain as the amyloid plaques are extracellular in patients (Cohen et al., 2006).

The recent failures of numerous anti-amyloid strategies and a strong correlation between tau tangles and disease progression in AD patients has motivated a renewed focus on tau toxicity (Haass and Selkoe, 2022). The intracellular nature of the neurofibrillary tangles raises the potential for activation of cellular pathways, such as the HSR, as therapeutic strategies. The fundamental nature of protein folding and the high levels of conservation of genes in the proteostasis network enable use of experimentally tractable model organisms. The metazoan nematode, *C. elegans*, has emerged as a powerful model organism to investigate neurodegenerative and other diseases (Caldwell, 2023; Dawson et al., 2018; Jiang and MacNeil, 2023). In this article we use *C. elegans* to explore the therapeutic potential of HSR activation for tau toxicity.

## RESULTS

**Fig. 1.**
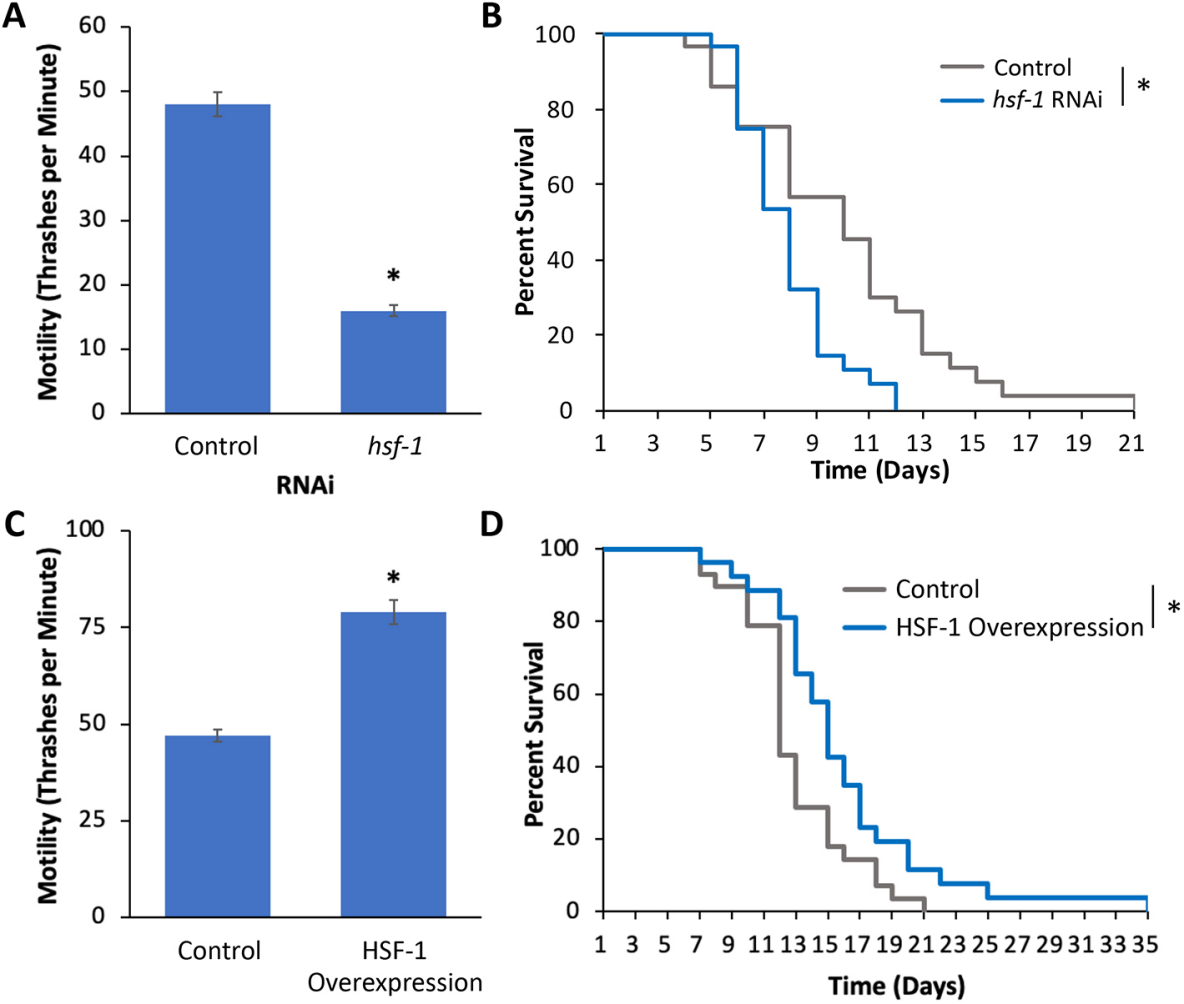
**HSF-1 affects motility and lifespan in a *C. elegans* tau model.** (A,B) Worms containing mutations that enhance RNAi and a human tau transgene were incubated on empty vector control (L4440) or *hsf-1* RNAi plates and assayed for motility by using a thrashing assay on day 1 of adulthood (A) and for lifespan by scoring for viability over time (B). Motility was significantly reduced in response to *hsf-1* RNAi. (C,D) Worms that contain the tau transgene, and those that contain the tau transgene and overexpress HSF-1 were incubated with bacteria grown on OP50 medium and assayed for motility (C) and for lifespan (survival in %) (D). Both the HSF-1 overexpression and the control strains lack mutations that enhance RNAi (C,D). Each bar represents the mean of *n*≥30 individuals. Error bars represent ±s.e.m.; *P-value<0.05; Student's *t*-test (A,C), log-rank test (B,D).

In this study, we investigated the role of the heat shock response (HSR) on tau toxicity to explore the therapeutic potential of this innate cellular defence pathway for AD and other tauopathies. We used *C. elegans* expressing the human tau isoform 4R1N comprising the P301S mutation (hereafter referred to as ‘tau’) in neurons. This disease model (hereafter referred to as ‘tau worms’) has a severe motility defect as measured using the thrashing assay in day-1 adults (Kraemer et al., 2003). To determine the effect inhibition of the HSR has on tau toxicity, we used RNA interference (RNAi) to knockdown *hsf-1*. The *hsf-1* gene encodes the HSF-1 transcription factor that mediates the HSR (Golden et al., 2020). This experiment was done in a strain containing two mutations, *eri-1*(*mg366*) and *lin-15B*(*n744*), both of which enhance the efficacy of RNAi, particularly in neurons (Kennedy et al., 2004). We found that *hsf-1* inhibition strongly exacerbated the tau-mediated motility defect, decreasing thrashes per minute from 48 in the control worms to 17 upon *hsf-1* inhibition (Fig. 1A). The effect was specific to disease model worms as *hsf-1* RNAi did not have a substantial effect on control worms (Fig. S1A). These results indicated that the HSR has a protective role against tau-mediated toxicity in early adulthood.

Tau toxicity also manifests in worms through a shortened lifespan. Therefore, we tested whether *hsf-1* can also exacerbate this aspect of tau toxicity. Tau worms subjected to *hsf-1* RNAi exhibited a shortened median lifespan of 8 days compared to 10 days with tau alone (Fig. 1B). However, this effect is not specific to tau, as *hsf-1* RNAi also shortened the lifespan of control worms containing only mutations that enhance RNAi (Fig. S1B). This finding is consistent with results from the previous literature indicating that protein aggregation is a normal feature of aging (Hsu et al., 2003). Together, these results are consistent with a protective role for *hsf-1* against tau-mediated toxicity.

Having established that HSR inhibition exacerbates tau toxicity, we next tested whether HSR activation is sufficient to ameliorate toxicity. For these experiments, we crossed the *C. elegans* tau model strain with an established strain that comprises HSR activation through overexpression of HSF-1 (EQ140) throughout the entire worm (Sural et al., 2019), therefore creating strain EAG29. We found that HSF-1 overexpression ameliorated the tau-mediated motility defect, increasing thrashes per minute from 47 in control worms to 79 in worms that contain the human tau transgene and also overexpress HSF-1 (Fig. 1C). The HSF-1-overexpressing strain contains a roller marker *(rol-6)* that did not contribute to the effect, as worms containing the tau transgene and an unrelated roller marker had 38 thrashes per minute (Fig. S1C). This effect is specific to tau as overexpression of *hsf-1* does not substantially increase motility in control worms (Fig. S1D). Additionally, HSR activation in response to HSF-1 overexpression increased the median lifespan from 12 days to 15 days in tau-only worms (Fig. 1D). However, this effect is not specific to tau, as overexpression of HSF-1 has previously been shown to increase lifespan in control worms (Sural et al., 2019). These results indicate that activation of the HSR ameliorates tau toxicity.

Having established that HSF-1 influences tau toxicity, we next tested whether regulators of the HSR also influence tau toxicity. Previously, we have identified a set of positive and negative HSR regulators in a genome-wide RNAi screen by using an HSR-dependent fluorescent reporter (Guisbert et al., 2013). Here, we performed a targeted RNAi screen of a representative subset of these regulators for their effects on motility in the tau model. At least one gene from each functional class was selected for analysis.

**Fig. 2.**
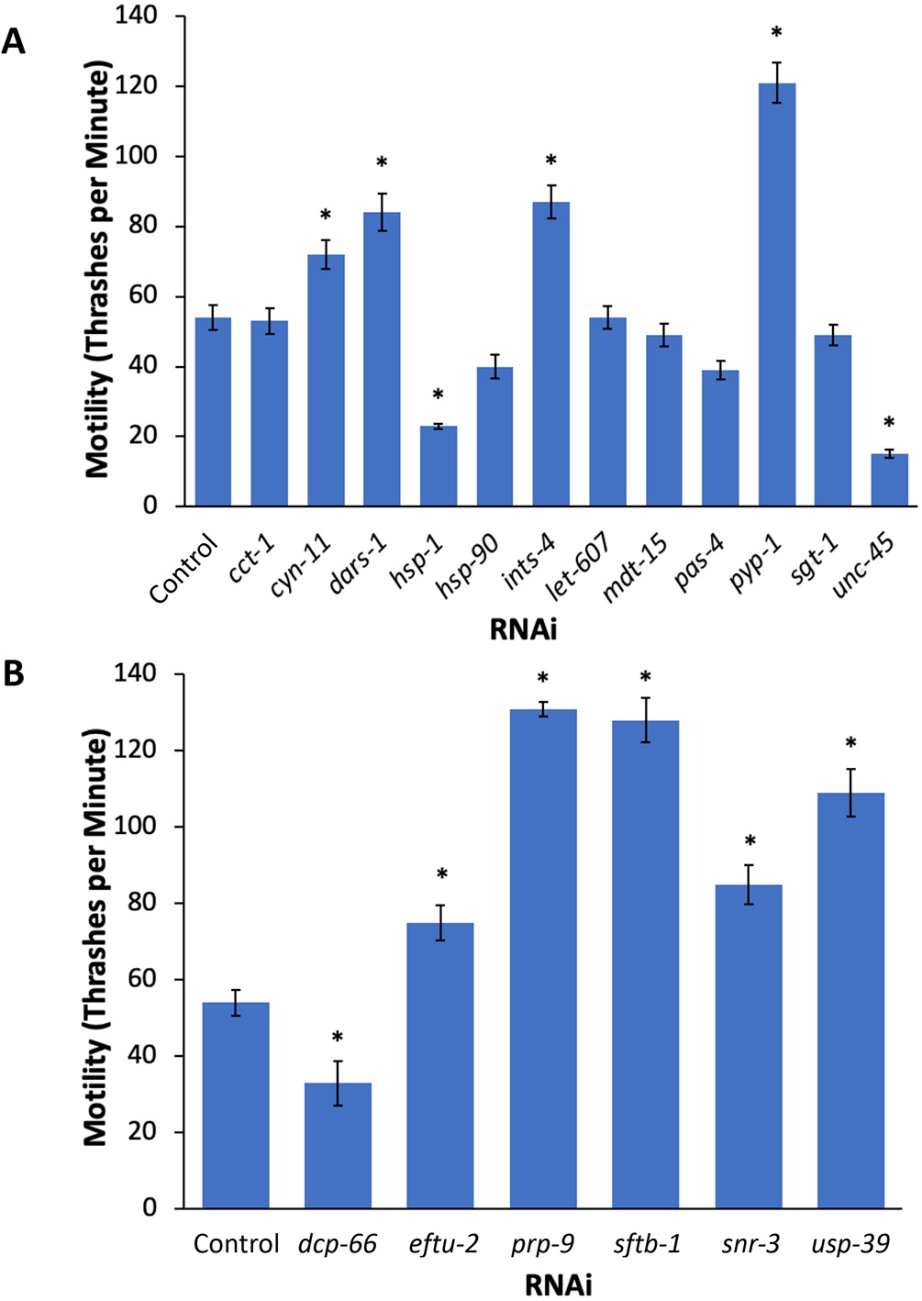
**HSR regulators affect tau toxicity.** (A,B) Worms containing the tau transgene and mutations that enhance RNAi were incubated on empty vector control (L4440) or RNAi plates seeded with bacteria targeting selected inhibitors (A) or activators (B) of the HSR. Motility was measured using a thrashing assay on day 1 of adulthood, showing that knockdown of *ints-4*, *pyp-1*, *dars-1* or *cyn-11* had the most-positive impact on motility (A), whereas knockdown of *dcp-66* (B) decreased mobility most. Each bar represents the mean of *n*≥30 individuals. Error bars represent ±s.e.m.; *P-value<0.05; one-way ANOVA.

As predicted, we found that RNAi of four negative regulators of the HSR (i.e. *ints-4*, *pyp-1*, *dars-1* or *cyn-11*) significantly ameliorated the motility defect of the tau model (Fig. 2A). The strongest effect – with more than doubled motility − came from *pyp-1*, a gene encoding an inorganic pyrophosphatase and a subunit of the nucleosome remodeling factor (NuRF) complex (Davis et al., 2022). The other three genes that positively influence tau toxicity encode a cochaperone (*cyn-11*), a tRNA synthetase (*dars-1*) and a subunit of the integrator complex (*ints-4*). The function of the integrator complex subunit is consistent with that described in the literature, showing that this complex coordinates RNA and protein quality control pathways (Wu et al., 2019). Discovery of HSR regulators that reduce tau toxicity expands the available targets for therapeutic strategies that activate the HSR.

In contrast, knockdown of two negative regulators, *hsp-1* and *unc-45*, were found to worsen the motility defect compared to control (Fig. 2A). Both of these genes have central roles in maintaining proteostasis, as they encode molecular chaperones that promote protein folding. Therefore, the enhancement of tau toxicity by these genes is consistent with their well-established cellular roles.

As expected, knockdown via RNAi of one positive regulator of the HSR (*dcp-66*), exacerbated the motility defect (Fig. 2B). *dcp-66* encodes a subunit of the nucleosome remodeling and deacetylase (NuRD) chromatin remodeling complex and has been shown to coordinate multiple stress responses (Golden et al., 2022). In contrast, five of the positive regulators of the HSR (encoded by *eftu-2*, *prp-9*, *sftb-1*, *snr-3* and *usp-39*) significantly improved motility in tau worms. *eftu-2* encodes elongation factor 2, *usp-39* encodes a deubiquitinase, and the proteins encoded by the other three are involved in mRNA splicing, including two subunits of the SF3 complex, i.e. SF3B1 and SF3A3 (encoded by *sftb-1* and *prp-9*, respectively), and the Sm core protein SMD1 (encoded by *snr-3*). While these regulators of HSR did influence tau toxicity, as predicted by our model, there was not always a correlation between their effects on HSF-1 activity and their effects on tau toxicity.

**Fig. 3.**
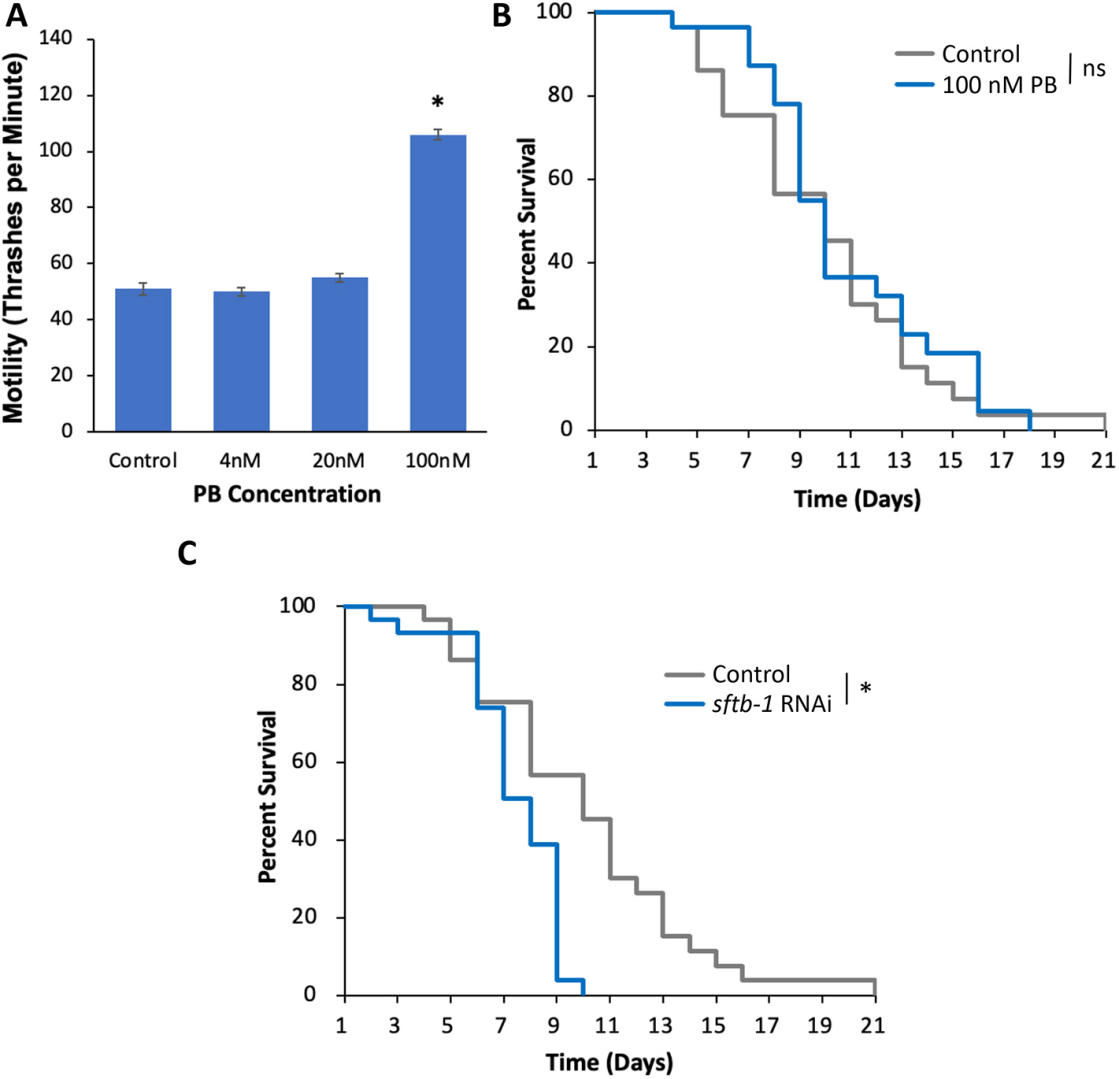
**SF3B1 ameliorates the tau-mediated motility defect but does not extend lifespan.** (A,B) Worms containing the tau transgene were incubated on control plates or plates containing the indicated concentrations of the SF3B1 inhibitor pladienolide B (PB) and assayed for motility (A) and lifespan (B). Motility (A) was measured using a thrashing assay on day 1 of adulthood, showing a significant increase of the tau-mediated motility defect in response to 100 nM PB (A). Lifespan (survival rate in %) (B) of untreated tau worms (Control, gray) and those treated with 100 nM PB (blue) was similar. (C) Tau worms containing mutations that enhance RNAi were incubated on empty vector control (L4440) or *sftb-1* RNAi plates and assayed for lifespan. Knockdown of *sftb-1* significantly reduced lifespan compared with that of control worms. Each datapoint represents the mean of *n*≥30 individuals. Error bars represent ±s.e.m.; *P-value<0.05; one-way ANOVA (A), log-rank test (B,C); ns, not significant.

Based on their effects, inhibition of *sftb-1* and other positive HSR regulators would be predicted to exacerbate tau toxicity; yet, we found that knockdown of this gene suppressed the motility defect (Fig. 2B). To validate these results, we tested the effects of the polyketide pladienolide B (PldB; also known and hereafter referred to as PB), a small-molecule inhibitor of SF3B1, encoded by *sftb-1* in *C. elegans* (Kim Guisbert and Guisbert, 2017). We found that similar to *sftb-1* knockdown, PB ameliorated the tau-mediated motility defect in a dose-dependent manner, increasing thrashes from 51 per minute to 106 (at 100 nM) (Fig. 3A). This improvement in motility was found to be tau specific, as an increase in motility was found in wild-type worms to a smaller extent (Fig. S2A). However, exposure to 100 nM PB did not increase lifespan of tau worms (Fig. 3B) or wild-type worms (Fig. S2B).

The effects of *sftb-1* inhibition on tau toxicity were also tested by analyzing the lifespan of worms. In this assay, knockdown of *sftb-1* through RNAi exacerbated tau toxicity, shortening median lifespan from 10 days to 8 days (Fig. 3C). This effect was also observed in worms without the tau transgene, with median lifespan shortened from 23 days for control versus 15 days for *sftb-1* RNAi (Fig. S1B). Therefore, these data indicate that either the beneficial effects of *sftb-1* inhibition are restricted to early adulthood or that they are offset by other detrimental effects that manifest during aging.

**Fig. 4.**
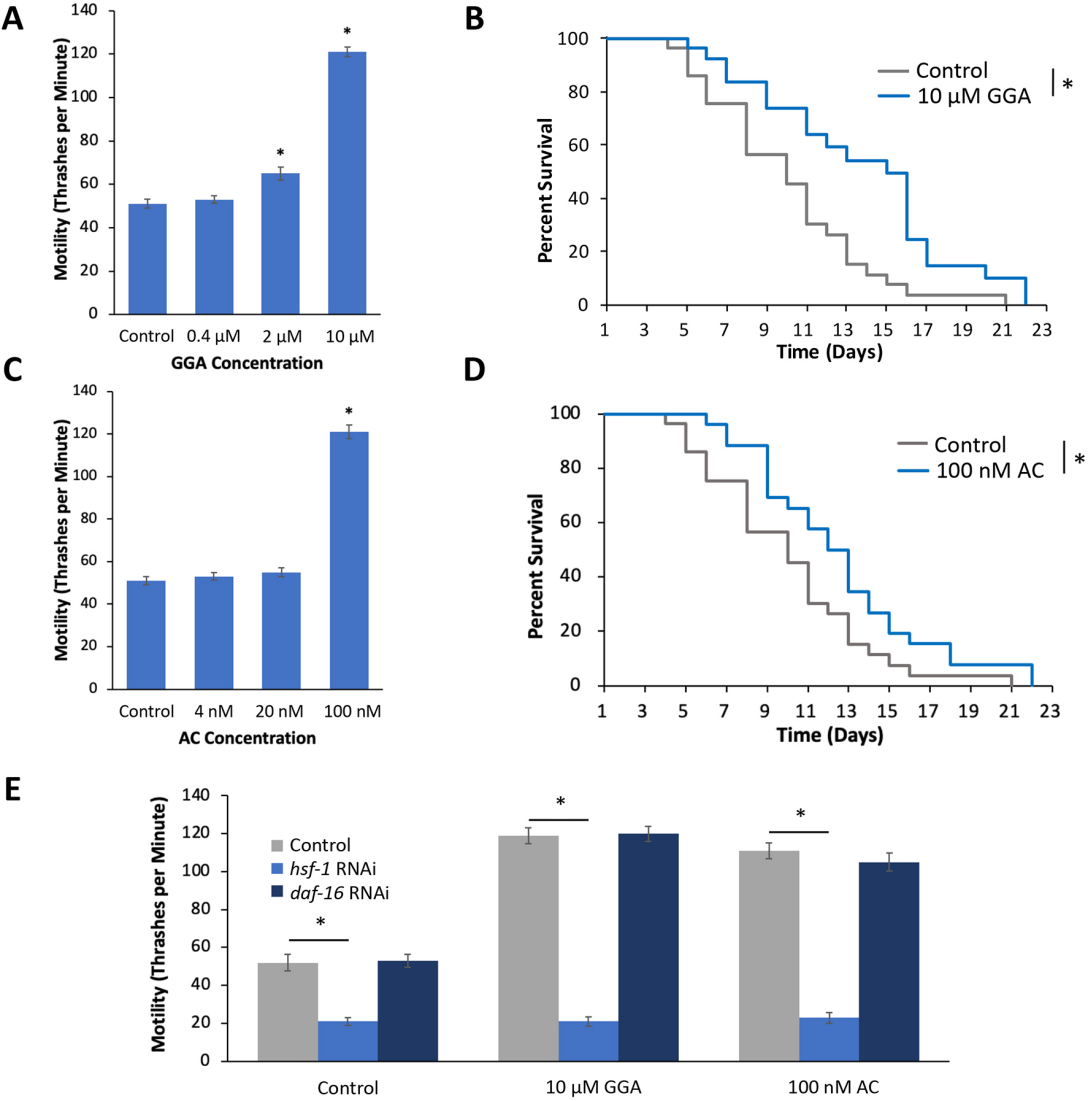
**Small-molecule activators of the HSR ameliorate tau toxicity in an HSF1-dependent manner.** (A-D) Worms containing the tau transgene were incubated on NGM (Control) plates, or plates containing the small-molecule HSR activators Geranylgeranylacetone (GGA) or Arimoclomol (AC) plates, and assayed for motility (A,C) and lifespan (B,D). At the highest concentration, both GGA and AC significantly increased motility and lifespan. (E) Worms containing mutations that enhance RNAi and the tau transgene were incubated on empty vector (Control), *hsf-1* RNAi or *daf-16* RNAi plates with and without GGA or AC, and motility was analyzed on day 1 of adulthood using thrashing assay. Knockdown of *hsf-1*, but not *daf-16*, significantly reduced the positive impact that GGA or AC had on the tau-mediated motility defect. Each datapoint represents the mean of *n*≥30 individuals. Error bars represent ±s.e.m.; *P-value<0.05; one-way ANOVA (A,C), log-rank test (B,D), two-way ANOVA (E).

As PB affected only the motility defect and did not extend lifespan, we next tested whether other small-molecule HSR activators can alter both phenotypes associated with tau toxicity. Geranylgeranylacetone (GGA) was approved for human use in Japan for the treatment of patients suffering from peptic ulcers and gastritis in 1984 (Grave et al., 2015). GGA has recently been shown to induce the HSR in *C. elegans* (Mossiah et al., 2022). The effects of GGA on tau toxicity were first measured by analyzing motility. GGA was found to ameliorate the tau-mediated motility defect in a dose-dependent manner. The highest dose of GGA (10 μM) showed the largest improvement in motility, increasing thrashes per minute from 51 in control worms to 121 in worms exposed to GGA (Fig. 4A). This effect was specific for tau worms as 10 μM GGA had only a minor effect on motility in N2 control worms (Fig. S3A). The 10 μM concentration of GGA also improved median lifespan in tau worms (10 days in control versus 15 days) (Fig. 4B). A beneficial effect of 10 μM GGA on median lifespan was also observed in wild-type worms albeit to a smaller extent (18 days in control days versus 20 days); however, this effect was not statistically significant (Fig. S3B). These results indicate that GGA alleviates both the motility defect and shortened lifespan due to tau-mediated toxicity.

AC is a hydroxylamine derivative that functions as a co-inducer of heat shock proteins (Elliott et al., 2020). AC is currently being considered for the treatment of multiple diseases including ALS. We first tested the effects of AC on tau toxicity by using the motility assay and found that, similar to GGA, AC also ameliorated the motility defect in a dose-dependent manner. At the highest concentration (100 nM), AC increased thrashes per minute from 51 in control worms to 121 (Fig. 4C). This improvement was specific for tau worms as 100 nM AC was found to increase motility by a smaller extent (Fig. S3C). Tau worms exposed to 100 nM AC were also shown to have an extended lifespan compared to tau worms on control plates, with median lifespan increasing from 10 days in control worms to 13 days (Fig. 4D). This lifespan extension was also observed in wild-type worms, with median lifespan increasing from 18 days to 20 days; however, this effect did not reach statistical significance (Fig. S3D). Therefore, we have identified two small-molecule activators of the HSR – GGA and AC − that can ameliorate both the motility defect and the lifespan defect caused by tau toxicity.

We next investigated the effects of GGA and AC when combined with HSF-1 inhibition, to determine whether the beneficial effects require an intact HSR. We found that *hsf-1* inhibition via RNAi reduced the effects of both GGA and AC on motility (Fig. 4E). Tau worms on *hsf-1* RNAi with either GGA or AC had thrashing rates of 21 and 23, respectively, versus 21 thrashes per minute on *hsf-1* control plates. This effect is specific as RNAi knockdown of *daf-16*, a component of the insulin-like signaling pathway that also controls lifespan and thermotolerance, did not diminish the effects of GGA and AC. These data indicate that GGA and AC against tau toxicity in an HSR-dependent manner.

We next investigated whether the protective effects of HSR activation occurred in a cell-autonomous manner in two complementary experiments. First, we tested the effects of HSF1 inhibition via RNAi in a strain lacking mutations that enhance RNAi, whereby efficiency of RNAi is slightly reduced throughout the worm but dramatically reduced in neurons (Kennedy et al., 2004). We found that *hsf-1* inhibition did not have a substantial effect on tau toxicity in worms lacking mutations that enhance RNAi (43 thrashes per minute versus 41 in control tau worms) (Fig. S4A). These data suggest that HSF-1 is acting in a cell-autonomous manner within neurons.

**Fig. 5.**
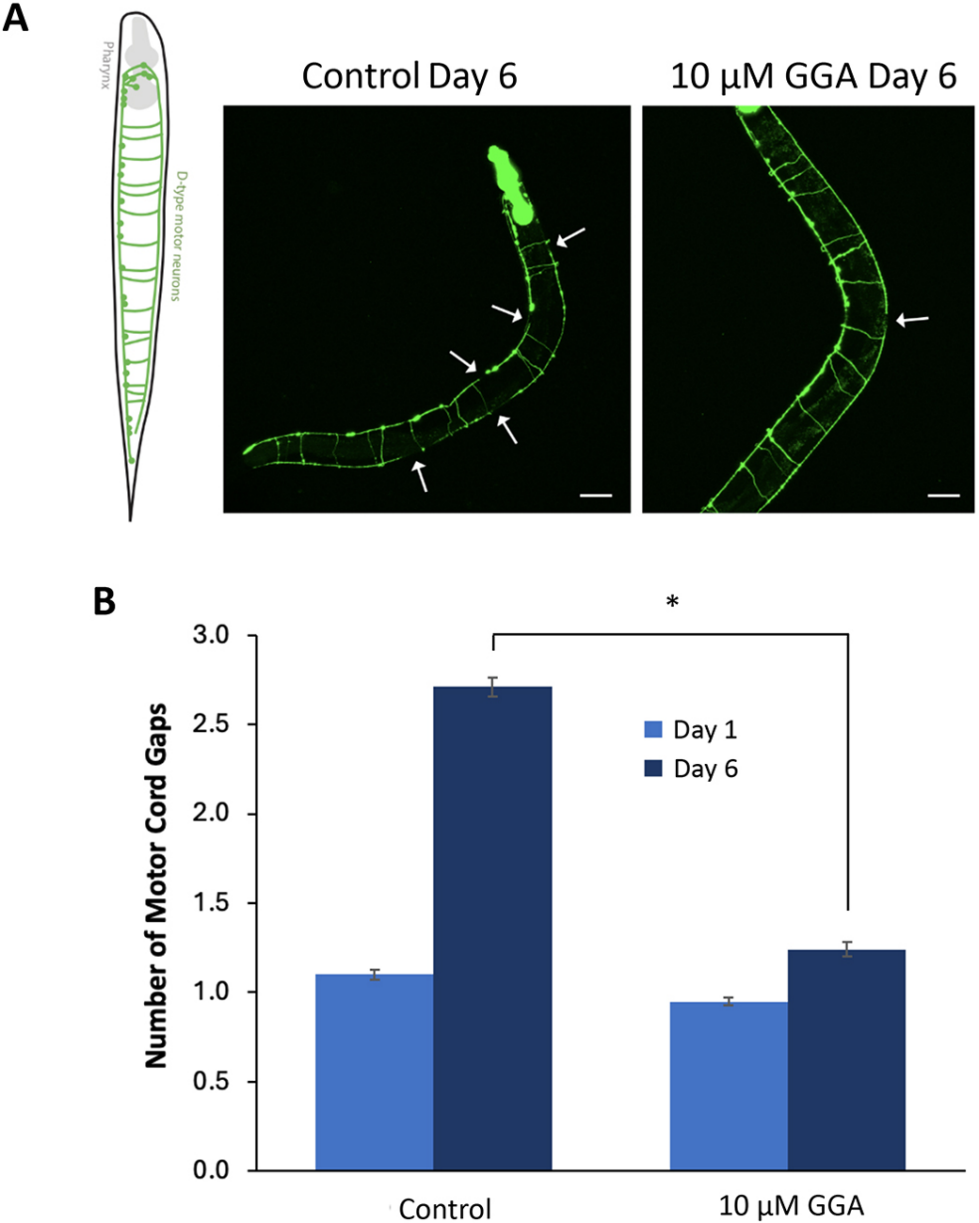
**GGA decreases motor cord gaps in a TauV337M model.** Worms containing the TauV337M transgene and a GFP neuronal marker were incubated on either NGM (Control) plates or on plates containing 10 μM Geranylgeranylacetone (GGA) and imaged on day 1 or day 6 of adulthood. (A) Left: Schematic of neurons in *C. elegans*. Right: Representative images of *C. elegans* not treated (Control) or treated with GGA. Images were taken at day 6 after treatment, showing that the number of motor cord gaps (indicated by arrows) are significantly reduced after treatment with GGA. (B) Bar graphs showing the average number of motor cord gaps in *n*≥70 individuals. Scale bars: 50 μm. Error bars represent ±s.e.m.; *P-value<0.05; two-way ANOVA.

We then tested whether the beneficial effects of HSR activation occurred in the neurons by directly visualizing neuronal integrity. For these experiments, we used a different tau model, Tau^V337M^, which has been shown to cause gaps in the motor nerve cord in day-6 adult worms that can be visually scored by using a neuronal GFP transgene (Fig. 5A) (Kraemer et al., 2003). We found that incubation of the Tau^V337M^ worms with GGA dramatically reduced the number of motor neuron breaks (1.2 vs 2.7 in control) (Fig. 5B). GGA also ameliorated the slight increase in motor nerve cord breaks due to aging in worms without the tau transgene (Fig. S4C). In contrast to the Tau^P301S^ model, the Tau^V337M^ worms only exhibited a mild motility defect, which was also reversed by treatment with GGA (Fig. S4B). Previous studies have indicated that mutations at these positions have similar effects when tau expression levels are normalized, suggesting that the reduced motility defect we observed in this strain is due to lower tau expression levels and not the specific mutation (Kraemer et al., 2003). Taken together, these data indicate that the beneficial effects of HSR activation extend to different tau mutations. Furthermore, at least part of these beneficial effects occur in a cell-autonomous manner within neurons.

## DISCUSSION

In this article, we demonstrate that the heat shock response pathway can modify tau toxicity in *C. elegans* by using both genetic and pharmacological approaches. Overexpression of the transcription factor HSF1 ameliorates tau toxicity in two complementary assays, motility and lifespan. By contrast, inhibition of *hsf-1* exacerbates this toxicity. Furthermore, genetic manipulation of several HSR regulators and two distinct small-molecule HSR activators can also influence tau toxicity. Direct visualization of motor nerve cord breaks in these models suggests that at least part of these effects occur cell autonomously. Taken together, these data support the further development of HSR-targeting therapeutics for the treatment of AD and other tauopathies.

Previously, it has been shown in *C. elegans* that HSR activation can combat protein aggregation and toxicity associated with the beta-amyloid peptide, although these two phenotypes are not always linked (Cohen et al., 2006; Gomez-Pastor et al., 2018). Our work shows the beneficial effects the HSR has on tau toxicity, which expands the therapeutic potential of the HSR in AD. The current amyloid cascade hypothesis suggests that both beta-amyloid and tau contribute to progression of AD. Therefore, the ability of the HSR to affect both types of proteotoxicity raises the possibility that HSR activation is beneficial in both early and later stages of the disease. Furthermore, there is a third feature of AD and other neurodegenerative diseases that can be influenced by the HSR, i.e. a general disruption of proteostasis, or protein folding homeostasis. Therefore, HSR activation has the potential to affect three distinct pathological features of the complex etiology of AD. It will be important in the future to further investigate these mechanisms.

HSR activation has also shown promise for the treatment of other neurodegenerative diseases. For example, AC, the small-molecule co-inducer of the HSR has shown promise in clinical trials for ALS and is currently undergoing further development. However, the full potential of HSR activation for treatment of neurodegenerative diseases remains to be determined. There are many distinct neurodegenerative diseases that each feature different proteins that misfold and aggregate. In humans, hundreds of different molecular chaperone genes function to prevent misfolding and aggregation. These chaperones interact in highly interconnected networks that feature extensive functional redundancy, making it difficult to anticipate the specific consequences of individual manipulations. As the HSR pathway has the ability to reinforce the proteostasis network generally through activation of a set of genes, it has the potential to influence many different types of protein misfolding. Since proteostasis disruption is a hallmark of neurodegenerative diseases distinct from pathological protein aggregation, HSR activation may be beneficial even if it is unable to prevent the primary pathological protein aggregation. In support of this, it has recently been shown that HSR activation via small-molecule regulators can restore tau-mediated disruption of endocytosis in a cell-culture model (Yu et al., 2019). However, it is important to explicitly test the hypothesis that HSR activation is beneficial for each specific diseases before this mechanism can be pursued as a therapeutic strategy.

Transcription factors, such as HSF-1, are particularly difficult to target with small molecules. Importantly, we have shown that genetic manipulation of several upstream regulators of HSF-1 also provide beneficial effects, expanding the potential space for therapeutic development. However, a known limitation of RNAi is that there can be variable levels of knockdown, making it difficult to interpret negative effects and make direct comparisons between genes (Kennedy et al., 2004). Interestingly, one of these factors, the splicing factor SF3B1, has also been identified in a screen for genes that prevent polyglutamine (polyQ) aggregation (Nollen et al., 2004). Inhibition of SF3B1 has been shown to promote aggregation in polyQ models but our work here indicates that it ameliorates toxicity in tau. One possible explanation for this difference is that the models are expressed in different tissues, muscle for polyQ and neurons for tau. It has been shown that many HSR regulators have tissue-specific effects (Ma et al., 2017). Yet, we have shown that inhibition of SF3B1 in response to high doses of the small molecule PB in cultured human cells also exacerbates polyQ aggregation (Kim Guisbert et al., 2020). However, lower doses of PB have the opposite effect, preventing polyQ aggregation. Similarly, different doses of PB have been shown to have distinct effects on HSF1 activation and HSF1 levels (Kim Guisbert and Guisbert, 2017). Therefore, it is clear that SF3B1 has a complex interaction with HSF1 and the proteostasis network. Unfortunately, in our current study, inhibition of SF3B1 only had a clear benefit on motility at the day-1 time point but was ultimately detrimental to lifespan in both the disease model and the wild-type controls, indicating that it may not be suitable for further development.

Thus far, no small molecule has been approved for use in humans with the goal of HSR activation. However, one small-molecule HSP activator that we tested, GGA, is an approved therapeutic in Japan and parts of Asia for the treatment of ulcers. AC, another small-molecule HSP activator that we tested, is currently being investigated in clinical trials. Thus, both of these molecules are bioavailable and have acceptable safety profiles for use in humans. Our results, showing that these molecules can ameliorate tau toxicity in a worm model, motivates further development of these molecules. Our work also supports identification of other compounds that can activate the HSR and provides a new model system for screening of compound libraries.

## MATERIALS AND METHODS

### Worm strains and maintenance

Standard laboratory techniques were used to maintain *Caenorhabditis elegans* nematodes at 20°C (Brenner, 1974). The *Escherichia coli* strain OP50 was used as the food source on Nematode Growth Medium (NGM) plates unless otherwise indicated. In all experiments, worms were age-synchronized by bleaching in a hypochlorite solution and hatching in M9 buffer overnight. The following strains were used: N2 (wild-type), CK1046 (*eri-1*(*mg366*); *lin-15B*(*n744*); bkIs1046 [*aex-3*p::tau 4R1N P301S Tg+*elt-2*p::mCherry]) (this study), KP3948 (*eri-1*(*mg366*); *lin-15B*(*n744*) (Kennedy et al., 2004), EQ140 (iqIs37[pAH76(*hsf-1*p::*myc-hsf-1*)+pRF4(*rol-6*p::*rol-6(su1006)*)]) (Sural et al., 2019), EAG18 (bkIs1046 [*aex-3*p::tau 4R1N P301S Tg+*elt-2*p::mCherry]), EAG29 (bkIs1046 [*aex-3*p::tau 4R1N P301S Tg+*elt-2*p::mCherry]); (iqIs37[pAH76(*hsf-1*p::*myc-hsf-1*)+pRF4(*rol-6*p::*rol-6(su1006)*)]) (Sural et al., 2019), CL2070 (dvIs70[*hsp-16.2p*::GFP+*rol-6(su1006)*]) (Link et al., 1999), EAG32 (bkIs1046 [*aex-3*p::tau 4R1N P301S Tg+*elt-2*p::mCherry]); (dvIs70[*hsp-16.2p*::GFP+*rol-6(su1006)*]) (Link et al., 1999). EAG33 (bkIs10 [*aex-3*p::tau 4R1N V337 M Tg+*myo-2*p::GFP]); (juIs76[*unc-25*p::GFP+*lin-15(+)*]) (Fatouros et al., 2012; Huang et al., 2002).

### Chemicals and reagents

Arimoclomol (AC), Geranylgeranylacetone (GGA) and pladienolide B (PB) were obtained from Santa Cruz Biotechnology and added to autoclaved medium just before pouring. L1 larval worms were directly exposed to the chemical-containing plates after synchronization. Worms were fed with bacteria from the ‘RNAi feeding library’ (Kamath and Ahringer, 2003) expressing double-stranded RNA (dsRNA) targeting various genes as specified in the text and figures. Bacteria containing the L4440 plasmid were used as the vector control. For all RNAi experiments, synchronized L1 larval worms were first plated onto NGM plates with OP50 bacteria as the food source. Worms were then washed in M9 and moved to RNAi plates after 19 h to initiate gene knockdown through RNAi at L2/L3 stage.

### Lifespan assay

For lifespan assays, viability of worms grown at 20°C was scored starting at day 1 of adulthood. Worms were scored as dead if they lacked pharyngeal pumping and had no movement in response to gentle prodding. Worms were moved to fresh plates to separate adult worms from progeny as needed.

### Motility assay

On day 1 of adulthood, worms were individually placed into a 35 μl spot of M9 buffer on a microscope slide and allowed to acclimate for 30 s. Then, thrashes were counted for a period of 30 s. One thrash was defined as 50% deviation of the body of the worm from its center line and back (Kraemer et al., 2003). For strains containing the roller marker (*rol-6*), one thrash was defined as one wave motion that persisted down the worm's body length [adapted from Shang et al. (2021)].

### Axon breaks

Worms were grown on standard NGM or 10 μM GGA plates. Worms were mounted in 5 mM Levamisole on agarose pads and scored for gaps in motor cords on day 1 or day 6 of adulthood. Motor cords were visualized using the *juIs76* transgene (*unc-25*p::GFP) under 40× magnification with a Zeiss Axioscope 5. A minimum of seven sets comprising ten animals were scored for each genotype and age. Representative confocal images were obtained using a Nikon Eclipse Ti2 Confocal Microscope under 20× magnification.

### Data analysis

Statistical significance (*P*-value) was calculated using Student's *t*-test in Excel for pairwise comparisons or ANOVA in R version 4.4.0 for multiple comparisons (R Core Team, 2024). Lifespan data were analyzed using the log-rank test in OASIS 2 (Han et al., 2016). All raw data can be found in Table S1.

## Supplementary Material

10.1242/dmm.050635_sup1 Supplementary information

Table S1. Raw data relating to Figs 1-5 and Figs S1-S4.
