# Supplementary material for: Activation of the heat shock response as a therapeutic strategy for tau toxicity
Source: Dis Model Mech. 2024 Oct 1;17(9):dmm050635. doi: 10.1242/dmm.050635 (PMC11463952; doi:10.1242/dmm.050635)
Supplement: Supplementary information [file dmm-17-050635-s1.pdf]

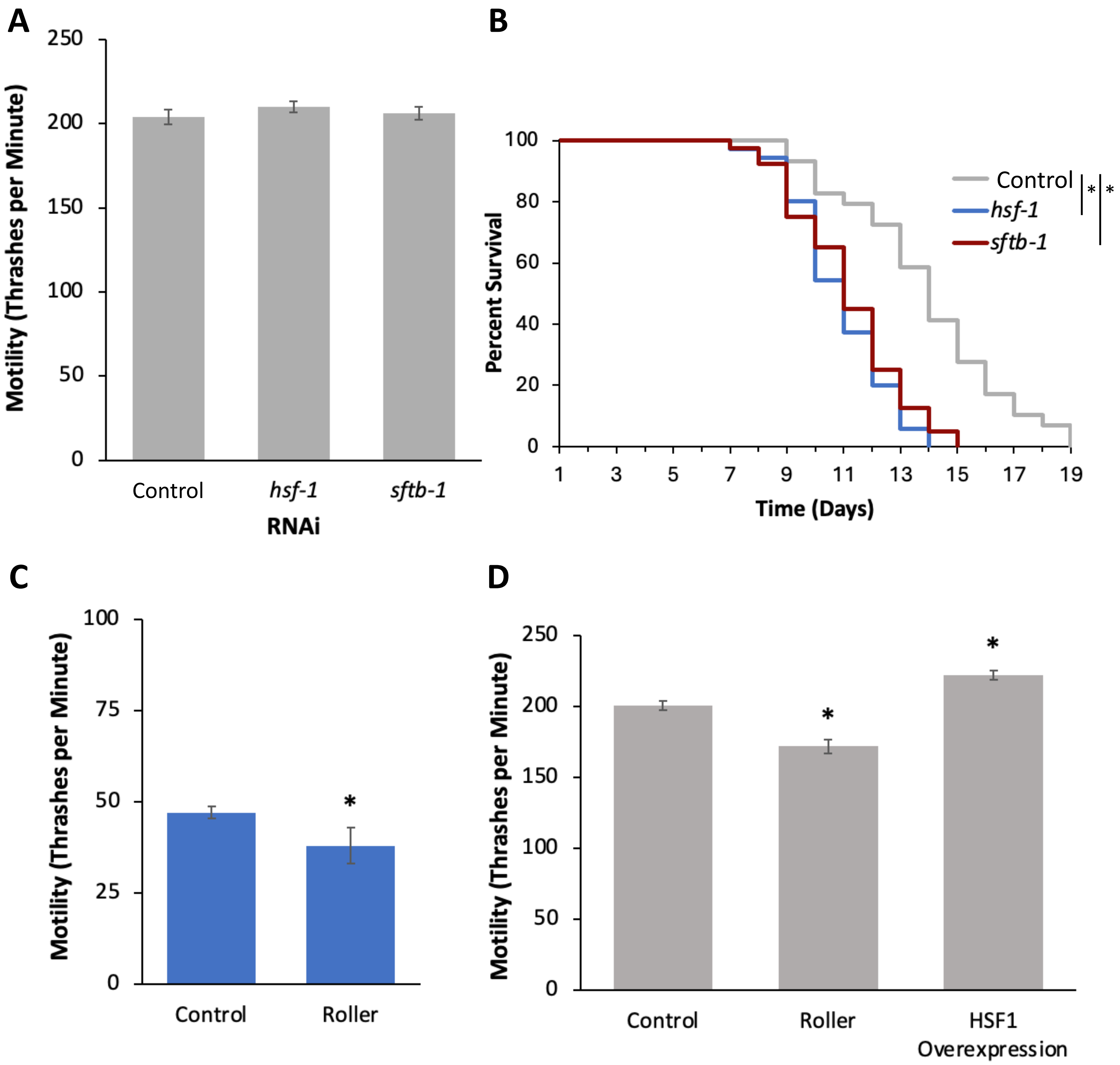

Fig. S1. HSF1 affects lifespan but not motility in non-tau worms. RNAi sensitized worms without the tau transgene were incubated on empty vector control (L4440), *hsf-1*, or *sftb-1* RNAi plates and assayed for motility using a thrashing assay on day 1 of adulthood (A) or lifespan (B). Non-sensitized worms with the tau transgene (Control) and worms containing the tau transgene and a *rol-6* marker but no *hsf-1* overexpression (Roller) were incubated on OP50 bacteria and motility was measured using a thrashing assay on day 1 of adulthood (C). Non-sensitized worms without the tau transgene (Control), worms containing a *rol-6* transgene but no *hsf-1* overexpression (Roller) or worms containing *rol-6* and *hsf-1* overexpression (HSF1 Overexpression) were incubated on OP50 bacteria and motility was measured using a thrashing assay on day 1 of adulthood (D). Each datapoint shown represents the mean of $n \geq 30$ individuals. Error bars represent s.e.m.; * indicates p-value < 0.05 (One-way ANOVA (A and D), log-rank test (B), and Student's *t*-test (C)).

Disease Models & Mechanisms • Supplementary information

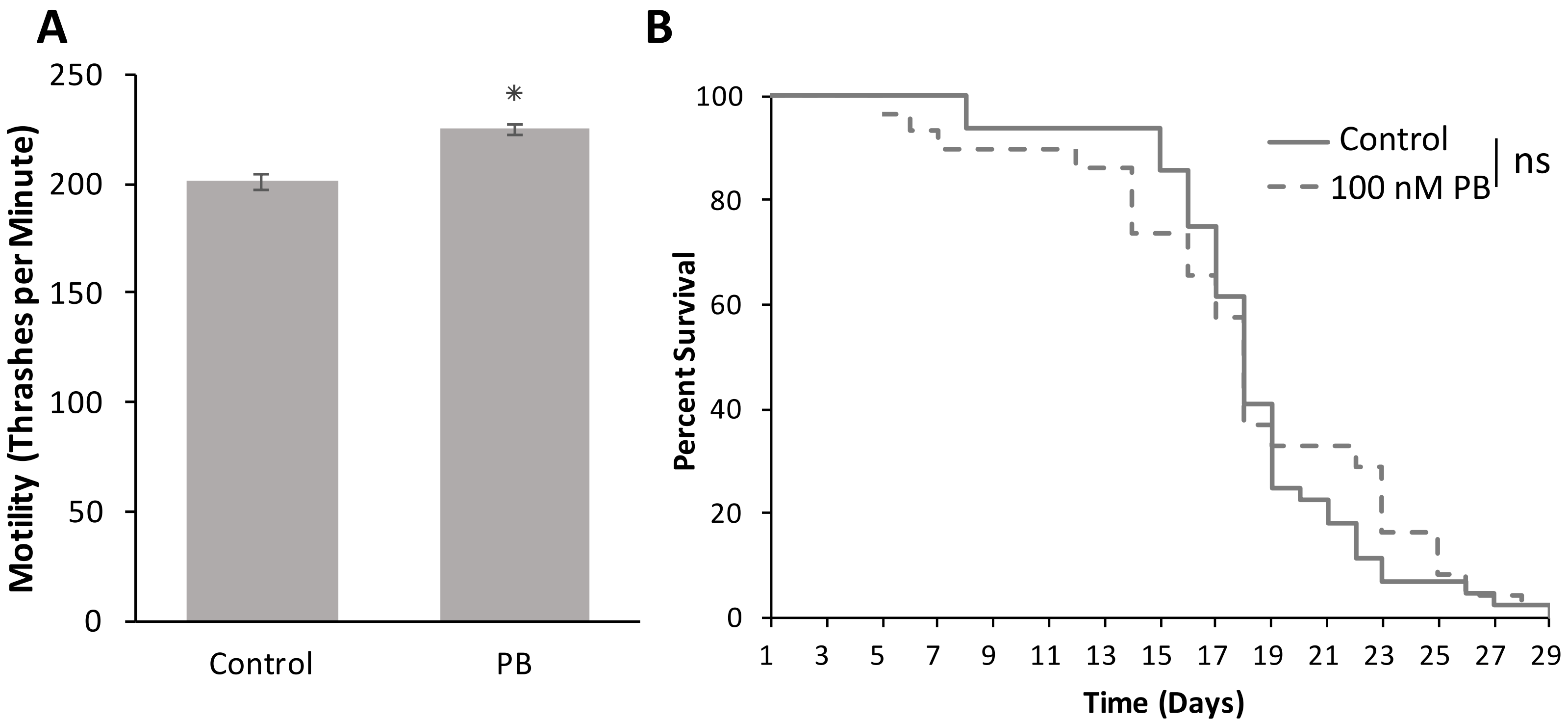

Fig. S2. SF3B1 does not extend lifespan in non-tau worms. Wild-type (N2) worms were incubated on either control plates or plates containing 100 nM pladienolide B (PB) and assayed for motility using a thrashing assay on day 1 of adulthood (A) and lifespan (B). Each datapoint shown represents the mean of n ≥ 30 individuals. Error bars represent s.e.m.; * indicates p-value < 0.05 (Student's *t*-test (A) and log-rank test (B)).

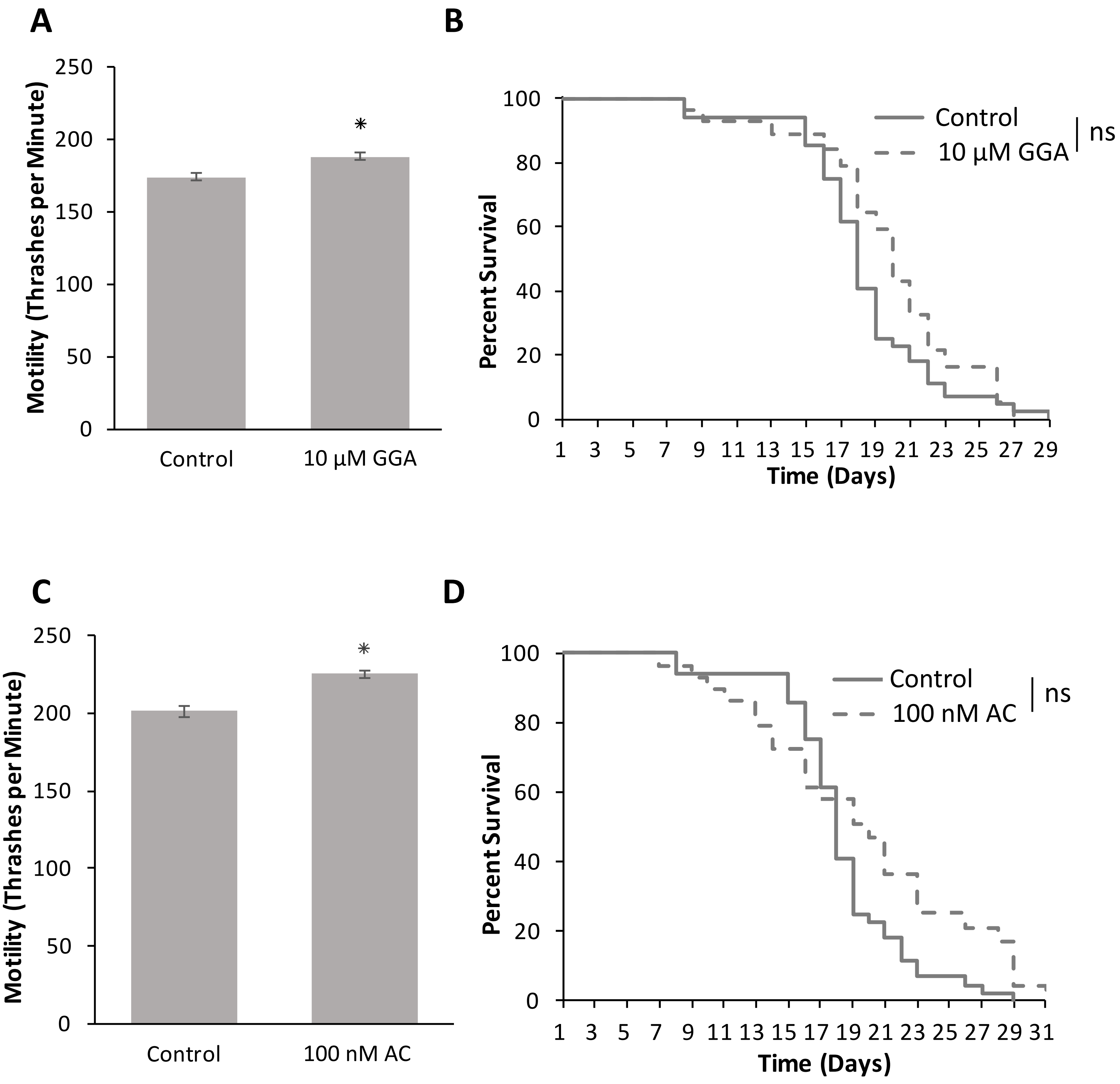

Fig. S3. The effects of small molecule activators of the HSR on lifespan in non-tau worms. Wild-type (N2) worms were incubated on either NGM control, 10 µM GGA, or 100 nM AC plates. Motility was measured using a thrashing assay on day 1 of adulthood (A & C) and viability was measured over time (B & D). Each datapoint shown represents the mean of n ≥ 30 individuals. Error bars represent s.e.m.; * indicates p-value < 0.05 (Student's *t*-test (A and C) and log-rank test (B and D)).

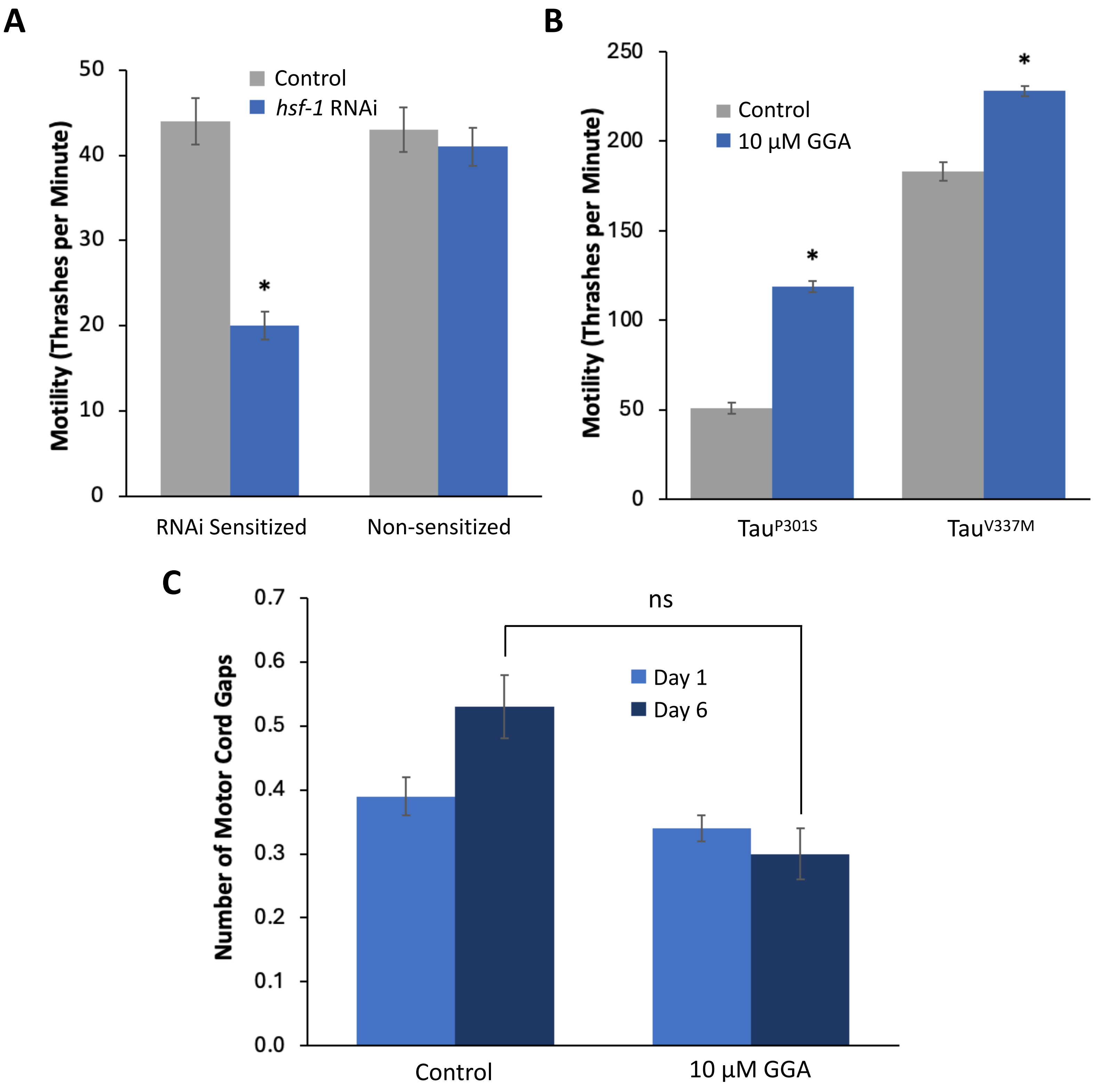

Fig. S4. HSF1 affects tau in a cell-autonomous manner. RNAi sensitized tau worms and non-sensitized tau worms were incubated on empty vector control (L4440) or *hsf-1* RNAi plates and assayed for motility using a thrashing assay on day 1 of adulthood (A). Tau^{P301S} and Tau^{V337M} worms were plated on either NGM control or 10 µM GGA plates and assayed for motility on day 1 of adulthood (B). Worms containing a GFP neuronal marker, but no tau transgene, were incubated on either NGM control plates or plates containing 10 µM GGA and motor nerve cord breaks were quantitated on day 1 or day 6 of adulthood (C). Each datapoint shown represents the mean of $n \geq 30$ individuals (A and B) or $n \geq 70$ individuals (C). Error bars represent s.e.m.; * indicates p-value < 0.05 (Two-way ANOVA).

Table S1. Raw data relating to Figs 1-5 and Figs S1-S4.

Available for download at
https://journals.biologists.com/dmm/article-lookup/doi/10.1242/dmm.050635#supplementary-data

Disease Models & Mechanisms • Supplementary information
